# Compliance of a ventilator-associated pneumonia care bundle in an adult intensive care setting

**DOI:** 10.1186/cc13196

**Published:** 2014-03-17

**Authors:** G Wigmore, R Sethuraman

**Affiliations:** 1 The Princess Alexandra Hospital NHS Trust, Harlow, UK

## Introduction

Ventilator-associated pneumonia (VAP) is the leading cause of death amongst hospital-acquired infections and is also linked to an increased length of stay and cost of care. The Institute for Healthcare Improvement ventilator bundle is comprised of a series of interventions, which, when implemented together, have been shown to decrease the incidence of VAP. The aim of this study was to determine the compliance of the bundle and if <95% [1] devise strategies to improve compliance.

## Methods

A retrospective review of the compliance of an existing VAP bundle was conducted for all adult patients ventilated in the ICU of a large district general hospital in 2011. The bundle comprised four elements: head up 30°, peptic ulcer prophylaxis, deep vein thrombosis (DVT) prophylaxis and sedation hold. The bundle was considered compliant if all four were performed. The findings of the audit were presented to the department and, through subsequent discussions, barriers to noncompliance were identified. Following a period of education, a revised and updated bundle was implemented. A repeat audit covering 3 months was subsequently conducted.

## Results

Pre-intervention, overall compliance of the bundle stood at 32% and subsequently increased to 63% post intervention (*P *< 0.05). Compliance at the level of individual elements varied: head up 30°, 94%; ulcer prophylaxis, 91%; DVT prophylaxis, 85%; sedation hold, 37%. Post intervention, a statistically significant increase in compliance with regard to sedation hold was observed; 72% (*P *< 0.05). The other individual elements did not show a statistical change. However, the new elements that were introduced demonstrated high levels of compliance; cuff pressure 20 to 30 cm H_2_O 83% and oral hygiene with chlorhexidine 90%.

## Conclusion

Post intervention, a statistically significant improvement in overall bundle compliance was found. Thereby highlighting that through engaging all members of the multidisciplinary team in identifying barriers to noncompliance and delivering education, it is possible to improve compliance. While total compliance was suboptimal as the target was 95%, the bundle redesign has given a tool that records compliance with greater clarity due to the presence of clearly defined exclusion criteria. It has also been a significant step in the right direction to improving the reliability of care delivered to patient and reinforces the concept that quality improvement is a continuous cycle.
